# Nuclear Receptor Subfamily 2 Group E Member 3 (NR2E3): Role in Retinal Development and Disease

**DOI:** 10.3390/genes14071325

**Published:** 2023-06-23

**Authors:** Maria Toms, Natasha Ward, Mariya Moosajee

**Affiliations:** 1 Development, Ageing and Disease, UCL Institute of Ophthalmology, London EC1V 9EL, UK; 2 Ocular Genomics and Therapeutics, The Francis Crick Institute, London NW1 1AT, UK; 3 Department of Genetics, Moorfields Eye Hospital NHS Foundation Trust, London EC1V 2PD, UK; 4 Department of Ophthalmology, Great Ormond Street Hospital for Children NHS Foundation Trust, London WC1N 3JH, UK

**Keywords:** NR2E3, inherited retinal disease, enhanced S-cone syndrome, retinitis pigmentosa, Goldmann–Favre syndrome, clumped pigmentary retinal degeneration

## Abstract

*NR2E3* is a nuclear hormone receptor gene required for the correct development of the retinal rod photoreceptors. Expression of NR2E3 protein in rod cell precursors suppresses cone-specific gene expression and, in concert with other transcription factors including NRL, activates the expression of rod-specific genes. Pathogenic variants involving *NR2E3* cause a spectrum of retinopathies, including enhanced S-cone syndrome, Goldmann–Favre syndrome, retinitis pigmentosa, and clumped pigmentary retinal degeneration, with limited evidence of genotype–phenotype correlations. A common feature of *NR2E3*-related disease is an abnormally high number of cone photoreceptors that are sensitive to short wavelength light, the S-cones. This characteristic has been supported by mouse studies, which have also revealed that loss of *Nr2e3* function causes photoreceptors to develop as cells that are intermediate between rods and cones. While there is currently no available cure for *NR2E3*-related retinopathies, there are a number of emerging therapeutic strategies under investigation, including the use of viral gene therapy and gene editing, that have shown promise for the future treatment of patients with *NR2E3* variants and other inherited retinal diseases. This review provides a detailed overview of the current understanding of the role of *NR2E3* in normal development and disease, and the associated clinical phenotypes, animal models, and therapeutic studies.

## 1. Introduction

*NR2E3 (*Nuclear Receptor subfamily 2 group E member 3; OMIM #604485), previously known as *PNR*, encodes a photoreceptor-specific orphan nuclear hormone receptor essential for the normal development of the retinal photoreceptors [1]. The gene is located on chromosome 15q23 and is comprised of 8 coding exons. *NR2E3* has two isoforms: (i) a full-length transcript containing all 8 exons, producing a 410-amino acid (aa) protein, and (ii) a second transcript that retains intron 7, coding for a smaller 367-aa protein that lacks the region encoded by exon 8 [2]. Pathogenic variants in *NR2E3* show significant clinical heterogeneity and have been associated with a number of retinopathies, with a lack of clear genotype–phenotype correlations [1]. A common hallmark of *NR2E3*-related disease is an abnormally increased number of cone photoreceptors that are sensitive to short wavelength (blue) light, the S-cones, which has been evidenced by psychophysical, electrophysiological [3,4], and histopathological [5] examination of patients, and animal studies [6]. While there is still much to be uncovered, significant progress has been made toward understanding the role of *NR2E3* in retinal development and disease and, in recent years, toward the development of effective treatments through promising pre-clinical therapeutic studies.

## 2. NR2E3 Structure

The NR2E3 protein is a member of a large family of ligand-modulated transcription factors, the nuclear receptors. In the human genome, there are 48 nuclear receptors, which include endocrine, adopted orphan, and orphan receptors [7]. NR2E3 is an orphan receptor that shares a conserved structural organization with all nuclear receptors, consisting of several key regions: the A/B, DNA-binding, hinge, and ligand-binding domains [8,9]. In the N-terminus, the highly variable A/B domain comprises a ligand-independent activator function (AF-1). This is followed by the most conserved region, the DNA-binding domain, which consists of two Cys_4_ zinc fingers that contain a P-box, thought to allow the receptor to bind to unique DNA response element sites and regulate gene expression, and a D-box, proposed to be involved in protein–protein interactions. The hinge domain links the DNA-binding and ligand-binding domains and contains a nuclear localization signal that may overlap with the DNA-binding domain.

The C-terminal ligand-binding domain of nuclear receptors typically consists of 12 α-helices that fold into a conserved hydrophobic pocket where a ligand could bind to, which is unknown in the case of NR2E3. In addition to this ligand-dependent activator function (AF-2), the ligand-binding domain is also essential for homo- and heterodimerization. Tan et al. solved the crystal structure of the ligand-binding domain of NR2E3 in a ligand-free state and found that it has a dimeric arrangement, with each monomer being formed of a canonical antiparallel three-layer α-helical sandwich fold made up of 8 α-helices [7,10]. The ligand-binding pocket was found to be filled by the side chains of hydrophobic and aromatic residues and the AF-2 helix occupies the canonical cofactor binding site. It was concluded that the NR2E3 ligand-binding domain has an auto-repressed configuration.

## 3. NR2E3 Function

### 3.1. Rod and Cone Photoreceptor Differentiation

The appearance of *NR2E3* in evolutionary time is thought to coincide with the emergence of rod and cone photoreceptors [1]. Prior to this, early vertebrate ancestors had only one photoreceptor cell type, which is thought to have been more structurally similar to cones than to rods [11]. Rods and cones differ in several key aspects, including their shape, photopigments, distribution within the retina, and pattern of synaptic connection [12]. Typically, the human retina contains ~5% cones and ~95% rods [13]. Cones are found at the highest density in the macula, whereas rods are more concentrated around the peripheral retina. Rods contain a single type of visual pigment, rhodopsin, for high-sensitivity low-light vision [12]. In contrast, human cones contain one of three alternative pigments (S-, M-, and L-opsins) each, which respond to short (S), medium (M), and long (L) wavelengths (i.e., blue, green, red, respectively) for color and bright-light high-resolution vision. The S-cone photoreceptor cell population is typically the least prevalent of the photoreceptor cell subtypes, accounting for 5–10% of the cone mosaic [14]. S-cones morphologically differ from M and L cones by displaying a longer and wider inner segment joining the outer segment and are most dense at ~2000 cells mm^2^, just outside the center fovea [15].

**Figure 1:**
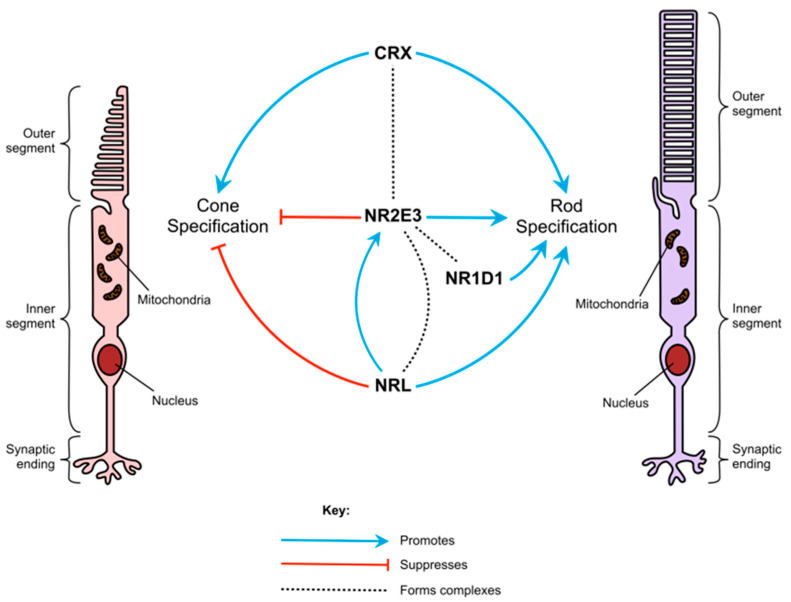
The role of *NR2E3* in photoreceptor cell specification. A schematic representation of how NR2E3 and other transcription factors interact to regulate rod and cone photoreceptor specification.

During embryonic development, rods and cones differentiate from common photoreceptor precursor cells [5]. Their differentiation is controlled by several transcription factors, including NR2E3, which ensure that rod- and cone-specific genes are confined to their corresponding photoreceptor type (Figure 1) [16]. Studies of animal models and human patients with *NR2E3* variants suggest that the gene’s role in photoreceptor differentiation is two-fold, in that it suppresses the expression of cone-specific genes, such as *OPNSW1* (blue opsin), *GNAT2,* and *GNB* (cone transducin subunits), and helps to activate the expression of rod-specific genes, such as the rod transducin β subunit *GNB1* [17] and rhodopsin [18,19]. NR2E3 is exclusively expressed in rods and is first detected in immature rods on the foveal edge from fetal week 11.7 [20]. Without the expression of *NR2E3*, photoreceptor precursors differentiate to the “default” photoreceptor cell type, S-cones [18].

Other than *NR2E3*, the main factors involved in rod differentiation are *CRX*, *NRL,* and *NR1D1* [16,21]. Evidence suggests that the transcription factors encoded by these genes, along with NR2E3, interact to form multi-protein transcriptional regulatory complexes [21]. CRX promotes the expression of both rod- and cone-specific transcripts, and pathogenic variants in *CRX* can cause several retinopathies, including early-onset diseases such as Leber congenital amaurosis [22] and cone-rod dystrophy [23]. Both NRL and NR2E3 are confined to rods and rod precursors and suppress cone-specific transcripts [1]. NRL also up-regulates the expression of NR2E3 and other rod-specific transcripts. Deletion of *Nrl* in mice also causes photoreceptors to develop as S-cones rather than rods [19].

Neither NR1D1 nor NR2E3 alone has much effect on the activity of rod-specific promoters [21]. However, when both are active together, NR1D1 and NR2E3 work synergistically to increase rhodopsin promoter activity. NR2E3 can also only bind to the promoter regions of rod-specific genes in the presence of CRX [24]. These interactions indicate that NR2E3 forms part of a complex network of signals that determine the cell fates of photoreceptor precursors. This complexity may in part account for the extensive variety of phenotypes seen in patients and animal disease models.

### 3.2. Role in the Adult Retina and Other Tissues

NR2E3 continues to be expressed in the adult retina, and several studies have indicated that it is involved in retinal maintenance [17,25,26]. In the mature mouse retina, NR2E3 regulates a different set of genes to those targeted in development; these include several genes responsible for the maintenance and survival of photoreceptors, such as phototransduction-related genes *Opnsw1* and *Gnb1* [14]. Furthermore, Olivares et al. found that *Nr2e3* is involved in several gene networks in the adult retina and regulates genes associated with age-related macular degeneration, including *Flt1*, *Abca1,* and *Alcam* [25].

Nr2e3 protein expression has been found in murine liver cells, suggesting its role is more widespread than was previously considered [27]. However, the role of NR2E3 in the liver and other tissues is not well understood. Higher levels of NR2E3 were associated with good clinical outcomes in liver cancer patients [28], while loss of NR2E3 was correlated with the development of liver disease and cancer [27]. Expression of NR2E3 showed a similar association in breast cancer patients, where it appears to regulate the estrogen receptor α [29].

## 4. Clinical Phenotype

**Figure 2:**
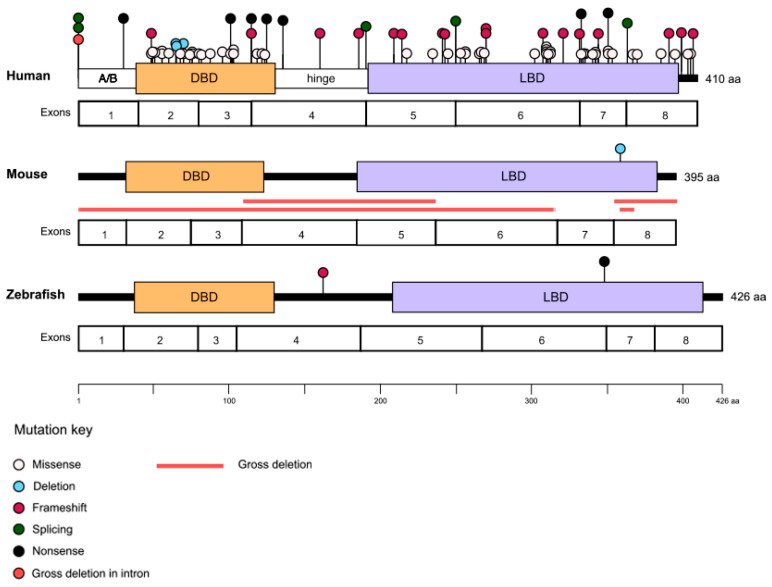
Pathogenic variants in *NR2E3*. The types and locations of the variants identified to date are marked on the human, mouse, and zebrafish NR2E3 proteins with the corresponding exons displayed underneath. Approximate locations of the A/B domain, DNA binding domain (DBD), hinge domain, and ligand binding domain (LBD) are marked on the human protein.

There are currently more than 80 identified disease-causing variants of *NR2E3*, which cause a variety of retinopathies, most of which show autosomal recessive inheritance (Figure 2 and Table 1). Among the recessively inherited disorders are enhanced S-cone syndrome (ESCS; MIM #268100), Goldmann–Favre syndrome (GFS; MIM #268100), retinitis pigmentosa (RP; MIM #611131), and clumped pigmentary retinal degeneration (CPRD). In addition, *NR2E3* is associated with an autosomal dominant form of RP.

**Figure 3:**
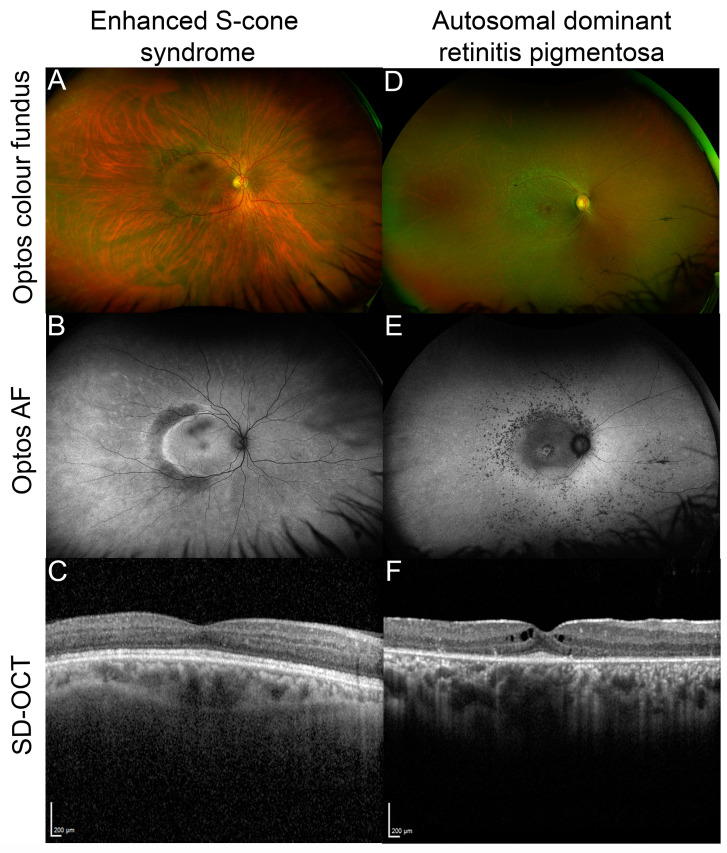
Examples of *NR2E3* patient retinal phenotypes. (**A**) Optos color fundus image of enhanced S-cone syndrome with (**B**) corresponding optos fundus autofluorescence (FAF) image showing diffuse peripheral hypoautofluorescence with a half-ring of pronounced hyper-AF along the temporal macular rim and (**C**) spectral domain optical coherence tomography (SD-OCT) through the macula of the same patient. (**D**) Optos color fundus image of autosomal dominant *NR2E3*-related retinitis pigmentosa with (**E**) corresponding optos FAF image that shows nummular hypoautofluorescent areas around the arcades, a hyperAF ring at the macula and another more diffuse ring along the arcades, and (**F**) SD-OCT through the macula of the patient showing cystoid macular edema and a restricted ellipsoid zone.

Pathogenic variants in *NR2E3* were initially described in patients with ESCS, a developmental condition that causes enhanced sensitivity to blue light, early onset night blindness (nyctalopia), and abnormal ERG responses due to an overabundance of S-cones and lack of functional rods [30]. Patients also have varying degrees of sensitivity to green and red light (due to varying abundance of M-cones and L-cones). Visual function for patients with these variants is highly variable, even within families, and can range from normal to severely reduced [31]. Additional clinical findings include hypermetropia, astigmatism, macular holes, vessel attenuation, and degenerative changes including subretinal white dots or yellow flecks and characteristic clumped or nummular pigment deposition observed in the mid-peripheral fundus [31,32,33]. Retinal images taken from a patient with ESCS are shown in Figure 3A–C. GFS is similar to ESCS, and the disorders are now considered to share the same clinical spectrum [33,34], with GFS representing a more severe form. In addition to enhanced S-cone function, early onset nyctalopia, and clumped fundus pigmentation, GFS is typically characterized by degenerative changes in the vitreous humor, macular and peripheral retinoschisis (splitting of retinal layers), and posterior subcapsular cataracts [35]. CPRD is a further disorder discovered to be on the *NR2E3* phenotypic spectrum, sharing some clinical features with ESCS and GFS (i.e., clumped pigmentation throughout the mid-peripheral fundus and nyctalopia early in life), with ERG responses more similar to that of RP patients [33,36]. However, an assessment of 11 confirmed *NR2E3* patients with CPRD, ESCS, or GFS revealed functional defects of little or absent rod function, regardless of diagnosis [33]. The same study found that pathogenic *NR2E3* variants accounted for approximately half of CPRD cases. In all *NR2E3*-associated disorders, there is degeneration of photoreceptors over time and patients often suffer from a progressive decline in vision [37,38]. However, follow-up assessments of some 50 patients have found that the best-corrected visual acuity remains stable over time for many patients [31]. In this study, the follow-up time ranged from 0 to 34 years, with a mean of 6.1 years.

ESCS, GFS, and CPRD are all linked to shared recessive biallelic *NR2E3* variants, and patients show considerable clinical heterogeneity even when carrying identical mutations [39]. The majority of the *NR2E3* pathogenic variants are found in either the DNA- or ligand-binding domains of the protein (Figure 2) [8,40]. For instance, the most common variant of *NR2E3* found in patients is a missense mutation in exon 6, c.932G>A p.(Arg311Gln), which occurs in the ligand-binding domain [30,33]. However, there are exceptions, including one of the most common *NR2E3* variants reported in the U.S., c.119-2A>C, which falls within the canonical splice acceptor site of intron 1 and has been shown to induce skipping on exon 2 [41]. It has not been possible to establish clear genotype–phenotype correlations among the recessively inherited *NRE23* diseases [8,33,39,41,42]. A number of factors may contribute to the high phenotypic variability, including the complex interactions between NR2E3 and other molecules involved in photoreceptor cell fate determination, the presence of modifier genes, and environmental influences.

*NR2E3* variants have also been identified as causing both autosomal recessive [43] and autosomal dominant RP [44]. RP is a common form of inherited retinal disease characterized by progressive loss of rod photoreceptors (presenting with nyctalopia and peripheral field loss) with subsequent cone degeneration, causing loss of central vision. In *NR2E3*-RP patients, night blindness is usually the first reported symptom starting in childhood or adolescence [43,44]. In typical RP, bone spicule-like pigment deposits are seen in the mid-peripheral retina; however, in some patients, clumped pigmentation has also been observed [37,43,45]. Retinal images taken from a patient with *NR2E3*-related autosomal dominant RP are shown in Figure 3D–F.

**Table 1:** Published patient *NR2E3* variants and associated phenotypes.

Region	Variant	Mutation Type	Amino Acid Change	Protein Domain	Reported Phenotypes	References
Intron 1	c.119-2A>C	Splicing			ESCS, GFS, CPRD, RP	[30,33,41]
	c.119-3C>G	Splicing			ESCS	[46]
	c.119-57_166	Gross deletion			RP	[47]
Exon 1	c.95G>A	Nonsense	p.Trp32 *	A/B	RP	[48]
Exon 2	c.142C>T	Missense	p.Arg48Cys	DBD	ESCS	[40]
	c.143_144delGCins25	Indel/ Frameshift	p.Arg48Glufs*66	DBD	RP	[49]
	c.145G>A	Missense	p.Val49Met	DBD	ESCS	[46]
	c.151G>A	Missense	p.Gly51Arg	DBD	ESCS	[40]
	c.166G>A	Missense	p.Gly56Arg	DBD	RP	[44]
	c.166G>C	Missense	p.Gly56Arg	DBD	RP	[50]
	c.188C>A	Missense	p.Ala63Asp	DBD	Cone–rod dystrophy	[51]
	c.194_202del	Deletion	p.Asn65_Cys67del	DBD	RP, ESCS	[30,52]
	c.196_201delGGCTGC	Deletion	p.Gly66_Cys67del	DBD	ESCS	[53]
	c.202A>G	Missense	p.Ser68Gly	DBD	ESCS	[54]
	c.211T>C	Missense	p.Phe71Leu	DBD	ESCS	[31]
	c.211_213delTTC	Deletion	p.Phe71del	DBD	ESCS	[42]
	c.223G>A	Missense	p.Val75Ile	DBD	RP	[55]
	c.226C>T	Missense	p.Arg76Trp	DBD	ESCS	[30]
	c.227G>A	Missense	p.Arg76Gln	DBD	ESCS	[30]
	c.242A>G	Missense	p.Tyr81Cys	DBD	ESCS	[46]
Exon 3	c.248G>A	Missense	p.Cys83Tyr	DBD	ESCS	[56]
	c.263G>T	Missense	p.Gly88Val	DBD	ESCS	[57]
	c.290G>A	Missense	p.Arg97His	DBD	ESCS, RD	[30,58]
	c.305C>A	Missense	p.Ala102Asp	DBD	ESCS, RP, RD	[59,60,61]
	c.309C>A	Nonsense	p.Cys103 *	DBD	RP	[62]
	c.310C>T	Missense	p.Arg104Trp	DBD	ESCS	[30]
	c.311G>A	Missense	p.Arg104Gln	DBD	ESCS, RP, RD	[63,64,65]
	c.328C>T	Nonsense	p.Gln110 *	DBD	RD, RP	[16,66]
	c.328dupC	Insertion/ Frameshift	p.Gln110Profs*31	DBD	GFS	[67]
Exon 4	c.352G>A	Missense	p.Val118Met	DBD	RP	[68]
	c.364C>T	Missense	p.Arg122Cys	DBD	RP, RD	[16,69]
	c.371C>T	Missense	p.Pro124Leu	DBD	RP	[70]
	c.373C>T	Nonsense	p.Arg125 *	DBD	ESCS, RP	[71,72]
	c.406G>T	Nonsense	p.Glu136 *	DBD	RP	[66]
	c.481delA	Deletion/ Frameshift	p.Thr161Hisfs*18	Hinge	RP, ESCS	[57,73]
	c.554delA	Deletion/ Frameshift	p.Lys185Serfs*66	Hinge	RP	[74]
Intron 4	c.571+2T>C	Splicing			RP	[75]
Exon 5	c.626dupA	Insertion/ Frameshift	p.Tyr209 *	LBD	RP	[74]
	c.639_640insT	Insertion/ Frameshift	p.Pro214Serfs*9	LBD	RD	[76]
	c.646G>A	Missense	p.Gly216Ser	LBD	GFS, RP	[72,77]
	c.701G>C	Missense	p.Trp234Ser	LBD	ESCS	[30]
	c.724_725delTC	Deletion/ Frameshift	p.Ser242Glnfs*17	LBD	ESCS, RP, cone–rod dystrophy	[78,79,80]
	c.731delT	Deletion/ Frameshift	p.Leu244Argfs*7	LBD	RP	[81]
	c.739C>T	Missense	p.Arg247Trp	LBD	ESCS	[31]
Intron 5	c.747+1G>C	Splicing			ESCS	[39]
Exon 6	c.755T>C	Missense	p.Leu252Pro	LBD	ESCS	[82]
	c.767C>A	Missense	p.Ala256Glu	LBD	ESCS, RD	[33,65]
	c.767C>T	Missense	p.Ala256Val	LBD	ESCS	[83]
	c.788T>C	Missense	p.Leu263Pro	LBD	ESCS	[57]
	c.790G>A	Missense	p.Gly264Arg	LBD	ESCS	[84]
	c.797T>A	Missense	p.Ile266Asn	LBD	RD	[61]
	c.808_809delCT	Deletion/ Frameshift	p.Leu270Alafs*70	LBD	ESCS	[85]
	c.827_843del17	Deletion/ Frameshift	p.Leu270Alafs*70	LBD	ESCS, GFS, CPRD	[33]
	c.908T>C	Missense	p.Leu303Pro	LBD	ESCS	[31]
	c.919_920delAT	Deletion/ Frameshift	p.Ile307Leufs*33	LBD	ESCS	[86]
	c.925C>G	Missense	p.Arg309Gly	LBD	ESCS	[30]
	c.925C>T	Missense	p.Arg309Trp	LBD	GFS	[87]
	c.926G>T	Missense	p.Arg309Leu	LBD	GFS	[72]
	c.926G>A	Missense	p.Arg309Gln	LBD	ESCS	[31]
	c.930C>G	Missense	p.Phe310Leu	LBD	ESCS	[88]
	c.931C>T	Missense	p.Arg311Trp	LBD	RD, RP	[66,89]
	c.932G>A	Missense	p.Arg311Gln	LBD	RP, ESCS, GFS, CPRD	[30,33,41,43]
	c.951delC	Deletion/ Frameshift	p.Thr318Argfs*6	LBD	RP	[90]
	c.967dupA	Insertion/ Frameshift	p.Met323Asnfs*18	LBD	RP	[52]
Exon 7	c.994G>A	Missense	p.Glu332Lys	LBD	ESCS	[59]
	c.994G>T	Nonsense	p.Glu332 *	LBD	RD	[91]
	c.1000C>G	Missense	p.Arg334Gly	LBD	ESCS	[63]
	c.1007T>C	Missense	p.Leu336Pro	LBD	ESCS	[57]
	c.1018G>A	Missense	p.Glu340Lys	LBD	ESCS	[92]
	c.1025T>C	Missense	p.Val342Ala	LBD	ESCS	[59]
	c.1025T>G	Missense	p.Val342Gly	LBD	RP	[93]
	c.1034_1038delTGCAG	Deletion/ Frameshift	p.Leu345 *	LBD	RP	[41]
	c.1048C>G	Missense	p.Gln350Glu	LBD	RD	[76]
	c.1048C>T	Nonsense	p.Gln350 *	LBD	ESCS	[94]
	c.1049A>G	Missense	p.Gln350Arg	LBD	ESCS, RP	[42,95]
	c.1057C>G	Missense	p.Leu353Val	LBD	ESCS	[57]
Intron 7	c.1101-1G>A	Splicing			ESCS	[46]
Exon 8	c.1112T>G	Missense	p.Leu371Trp	LBD	ESCS	[92]
	c.1118T>C	Missense	p.Leu373Pro	LBD	ESCS, RD	[16,96]
	c.1154G>C	Missense	p.Arg385Pro	LBD	ESCS	[30]
	c.1171_1172delTT	Deletion/ Frameshift	p.Phe391Profs*15	LBD	RP	[97]
	c.1184T>C	Missense	p.Ile395Thr	LBD	RP	[98]
	c.1194delT	Deletion/ Frameshift	p.Pro399Glnfs*79		ESCS	[59]
	c.1217A>G	Missense	p.Asp406Gly		GFS	[99]
	c.1220T>A	Missense	p.Met407Lys		ESCS	[30]
	c.1223delT	Deletion/ Frameshift	p.Phe408Serfs*7		RD	[16]

DBD, DNA-binding domain; LBD, ligand-binding domain; ESCS, enhanced S-cone syndrome; CPRD, clumped pigmentary retinal degeneration; GFS, Goldmann–Favre syndrome; RP, retinitis pigmentosa; RD, unspecified retinal dystrophy; * premature termination codon (PTC). The pathogenic variants are listed in the Human Gene Mutation Database (HGMD) Professional version, accessed on 16 January 2023.

There is a genotype–phenotype association for the autosomal dominant RP, with all cases linked to an *NR2E3* missense variant, c.166G>A p.(Gly56Arg), which occurs in the first zinc finger of the DNA-binding domain [44]. This variant has been found to cause 1–2% of autosomal dominant RP in North America [51] and to have a frequency of 3.5% in a large Spanish cohort [37]. Functional analysis showed that the absence of DNA-binding but competition for dimer formation may explain the dominant negative activity exhibited by the p.(Gly56Arg) mutant protein [45]. Furthermore, the p.(Gly56Arg) mutant NR2E3 protein was found to show a distinct in vivo protein–protein interaction with CRX, comparable to that of wild-type NR2E3, and unlike the protein with biallelic recessive variants located in the DNA-binding domain [100]. Escher et al. investigated a family with autosomal dominant RP caused by the heterozygous p.(Gly56Arg) variant, in which two family members that carried compound heterozygous variants (p.Gly56Arg/p.Arg311Gln) had an ESCS-like phenotype [45]; it was suggested that the p.(Arg311Gln) variant may have a beneficial modifying effect on p.(Gly56Arg) due to increased photoreceptor-specific gene expression caused by impaired corepressor binding.

## 5. Animal Models

One commonly used *NR2E3* animal model is the *rd7* mouse, which was initially considered to have a naturally occurring recessively inherited 380 bp deletion in the coding region of *Nr2e3*, resulting in a frameshift premature stop codon [101]. However, Chen et al. later reported in 2006 a 10-fold increase in a 9 kb photoreceptor-specific Nr2e3 transcript, which was found to arise from the antisense insertion of a long interspersed nuclear element (LINE-1) (or L1) into exon 5. This L1 insertion subsequently blocks splicing, leading to incompletely spliced transcripts and their accumulation of mutant Nr2e3 in photoreceptor nuclei [102]. These mice suffer from progressive photoreceptor degeneration starting at 12 months and have a 1.5 to 2-fold increase in S-cone numbers [1]. At the age of 1 month, the outer nuclear layer of *rd7* mouse retinas shows patterns of waves, whorls, and rosettes, which gradually disappear between 5 and 16 months [101]. ERGs are normal until 5 months, after which there is a progressive reduction in signals for both rods and cones. A mottled pigment can be seen in the retina at the age of 16 months, along with a reduction in outer nuclear layer thickness. The retinas of these mice also included some cells that are intermediates between rods and cones [1]. Cheng et al. found that 50% of cells expressing S-opsin in the *rd7* mouse also express Nrl, which is not seen in wild-type mice. In the *rd7* retina, cells that should develop into rods show downregulated expression of rod-specific genes (such as *Rho*, *Gnb1,* and *Pde6b*) and upregulated expression of cone-specific genes (such as *Opnsw*, *Gnb3,* and *Pde6c*) compared with those of wild-type [18].

A recent longitudinal study using spectral domain optical coherence tomography (SD-OCT) to compare the retinas of *rd7* mice and ESCS patients found that the disease progression correlates well between the two species, and identified characteristics on the patient scans that may be equivalent to the whorls and rosettes seen in mice [103].

An additional *Nr2e3* knockout mouse model (*Nr2e3^−/−^)* mouse was previously generated through the ablation of exons 1–6, showing a phenotype and gene expression profile similar to the *rd7* mouse [104]. In recent years, two new mouse models have been generated by Aísa-Marín et al. using the CRISPR/Cas9 D10A nickase system [2]. Allele Δ27 was an in-frame deletion of 27 bp in exon 8 that ablates the dimerization domain, whereas allele ΔE8 (full deletion of exon 8) produced only the short isoform, which lacks the C-terminal part of the LBD involved in repressor activity.

Both models showed retinal invaginations similar to the rosettes found in the *rd7* mouse; however, the ΔE8 model displayed an RP-like phenotype with progressive retinal degeneration, while Δ27 had a more ESCS-like disease with developmental defects.

**Figure 4:**
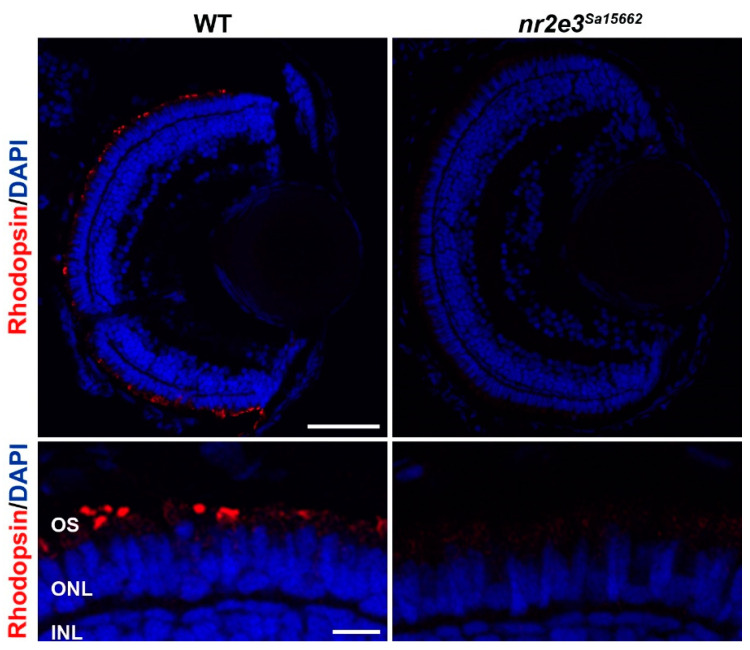
Lack of rod differentiation in *nr2e3* mutant zebrafish. Immunohistochemical staining for rhodopsin shows a lack of rod photoreceptors (red) in the *nr2e3^Sa15662^* mutant retina at 5 days post fertilization. Nuclei are counterstained with DAPI (blue). Scale bars are 50 µm (top) and 10 µm (bottom). Retinal layers are indicated: photoreceptor outer segments (OS), outer nuclear layer (ONL), and inner nuclear layer (INL). Image kindly prepared by Dr. Manuela Lahne from Prof. Mariya Moosajee’s group.

Zebrafish have been used to study the role of *NR2E3*. Xie et al. used CRISPR/Cas9 to create an *nr2e3* knockout line with a 37 bp deletion c.485_521del, causing a frameshift premature stop codon (p.Leu162Glnfs*30) [105]. These fish showed no rhodopsin expression and a lack of rod photoreceptors at 10 days post fertilization, which were still absent at ages 6 and 10 months. Other rod-specific genes, such as *gnat1* and *pde6b,* were not expressed at the mRNA or protein level. These fish were found to suffer from selective degeneration of L- and M-cones, with their outer segments beginning to shorten around the age of 1 month. However, the variant appeared to have no effect on the number of UV- or S-cones. The *nr2e3* Sanger zebrafish line *Sa15662* harboring a nonsense mutation in exon 6 (c.1036A>T, p.[Lys346*]) also shows an absence of rod photoreceptors (Figure 4) (unpublished data).

As in humans, the expression of *nr2e3*/*Nr2e3* in zebrafish and mice is largely confined to the photoreceptors, although in zebrafish the expression of *nr2e3* is transiently expressed in both rod and cone precursors during early photoreceptor development [105]. Other than the initially normal ERGs, the mouse *rd7* model appears to phenocopy human patients with *NR2E3* pathogenic variants in a fairly faithful manner [38]. The zebrafish model does not mimic the human disease as closely, as it does not show any increase in S- or UV-cone development. However, the lack of rod cell development and the progressive degeneration of L- and M-cones are reminiscent of human patients with *NR2E3* variants. The differences between human patients, *rd7* mice, and zebrafish *nr2e3* models are most likely due to the evolutionary history of rods and cones. Mammalian S-cones are thought to be most closely related to teleost UV-cones, whereas teleost M-cones are evolutionarily closer to the mammalian rods than cones [11].

## 6. Treatments

Although there are currently no approved therapies for the primary genetic defects associated with *NR2E3*, several strategies for treating *NR2E3*-related retinal disease and other inherited retinopathies are currently under development and have shown promise. The treatment strategy will likely depend on the clinical phenotype, with developmental effects on the ESCS spectrum, such as the low number or absence of rod photoreceptors, posing a greater challenge as the ideal window for therapeutic intervention may be prenatal. In contrast, the late onset of RP provides a longer and more accessible time period for potential intervention.

One of the leading treatment avenues being pursued for many inherited retinal diseases is the use of viral gene therapy for the replacement of the defective gene. A phase 1/2 clinical trial (NCT05203939) has commenced testing AAV-*NR2E3* gene therapy (OCU400) in adults with autosomal recessive and dominant *NR2E3* retinopathy. This is based on the work of Li et al. [26], who investigated the use of AAV vectors to over-express *Nr2e3* in 5 different mouse models of RP, via subretinal injection in neonatal mice or adult mice. This method was found to reduce retinal degeneration caused by mutations in several genes, as well as *NR2E3*, highlighting this as a potential broad-spectrum therapy for multiple retinopathies. In addition, subretinal delivery of *Nr1d1* using expression constructs was able to ameliorate retinal degeneration in the *rd7* mouse, further demonstrating the beneficial effects of modifier genes in mediating disease progression [106]. Interestingly, intravitreal injection of Nr2e3 antagonist photoregulin-1 has been shown to prevent photoreceptor death in rod degeneration mouse models Pde6brd1 and RhoP23H [107], with similar results being found in knockout experiments of Nrl in the adult retina [108]. This demonstrates the marked difference in roles of Nr2e3 in the developing versus adult retinas.

A potential agnostic gene therapy approach for patients suffering from advanced stages of RP is the use of optogenetics, which involves the transduction of light-sensitive ion channels called channel rhodopsin or related photosensitive molecules, which can open existing ion channels on the remaining retinal cells, including ganglion and bipolar cells [109,110]. Clinical trials are underway (NCT02556736; NCT03326336; NCT04945772). However, while these methods may restore some measure of light sensitivity to patients, they would be unlikely to result in high-resolution vision.

Preclinically, CRISPR/Cas9 gene editing was used to correct *NR2E3* pathogenic variants in induced pluripotent stem cells (iPSCs) generated from two patients with ESCS, using a homology-directed repair (HDR)-based strategy [111]. Patient 1 carried a homozygous c.119-2A>C splice site variant, and patient 2 had compound heterozygous p.(Arg73Ser) and p.(Arg311Gln) variants. They achieved relatively high efficiency (~72–83% of clones initially screened showed incorporation of HDR cassette) with minimal off-target mutagenesis. As most of the *NR2E3* pathogenic variants are point mutations or small deletions rather than large indels or complex rearrangements, CRISPR/Cas9 may be applicable to *NR2E3*-related diseases in the future.

The frequent autosomal dominant *NR2E3* RP-associated variant, p.(Gly56Arg), was targeted using antisense oligonucleotides (AONs), which were designed to bind and silence expression of the mutant mRNA transcripts by inducing RNAse-H1 cleavage [112]. Wild-type or mutant *NR2E3* was over-expressed in RPE-1 cells before treatment with the AONs, all of which showed a general knock-down in both the mutant and wild-type *NR2E3* at the mRNA and protein level, although a preferential mutant protein-specific knock-down was observed for most of the AONs. While this investigation showed the accessibility of the region for AON-induced knockdown, further modifications are needed to increase allele-specificity to ensure this is an effective therapeutic approach in the future.

Although it is unlikely that the gene-based therapies described would reverse developmental photoreceptor defects in adults with ESCS-type disease, *NR2E3* continues to be expressed in adult life, and boosting its function or that of its modifier genes with such treatments might help to maintain normal photoreceptor function and prolong survival. Alternatively, in patients with ESCS or late-stage RP, stem cell therapies to replace photoreceptor loss may be the most suitable future option [113,114]. However, it may not be possible for implanted cells to form proper synaptic connections in the retinas of patients with developmental defects.

While there is currently no approved treatment for genetic defects underlying *NR2E3* disorders, the associated disease complications, such as hypermetropia and retinoschisis, should be monitored and management offered where appropriate to reduce further sight loss. For instance, macular retinoschisis and cystoid macular edema can be effectively treated using the oral carbonic anhydrase inhibitor, acetazolamide [115,116].

## 7. Future Perspectives

In spite of the variety of studies conducted on *NR2E3* and its role in the developing and mature retina, there are still significant gaps in our understanding. The high levels of disease heterogeneity, particularly among patients with recessive mutations, demonstrate the complex interactions of this gene that have yet to be understood. A greater understanding of the underlying disease mechanisms and genotype–phenotype correlations is needed to better inform genetic counseling and the most effective treatment approaches. Further investigation into the function of *NR2E3* will be aided by the use of iPSCs, which have already been generated from patients with *NR2E3* pathogenic variants. iPSC-derived retinal organoids have recapitulated the enhanced S-cone phenotype and have been used for in vitro therapeutic studies [111,117,118,119]. For in vivo disease modeling, the CRISPR/Cas9 system could be used to create higher-order animal models harboring specific mutations, which can further aid in pre-clinical therapeutic work. In recent years, *NR2E3* has been highlighted as an important modifier of retinal disease and, in addition to the treatment of patients with *NR2E3*-related conditions, *NR2E3* gene supplementation could be pursued as a broad-spectrum therapy for various other retinopathies, which is particularly promiseng for the significant proportion of patients that remain without a molecular diagnosis.

## Data Availability

This is a review so previous data is referenced.
